# Effect of Cooling Rate on the Microstructure Evolution and Mechanical Properties of Iron-Rich Al–Si Alloy

**DOI:** 10.3390/ma15020411

**Published:** 2022-01-06

**Authors:** Xiao Shen, Shuiqing Liu, Xin Wang, Chunxiang Cui, Pan Gong, Lichen Zhao, Xu Han, Zirui Li

**Affiliations:** 1 School of Mechanical Engineering, Hebei University of Technology, Tianjin 300401, China; sx961216@163.com (X.S.); xhan@hebut.edu.cn (X.H.); lizirui@gmail.com (Z.L.); 2 State Key Laboratory for Reliability and Intelligence of Electrical Equipment, School of Materials Science and Engineering, Hebei University of Technology, Tianjin 300401, China; 3 Key Laboratory for New Type of Functional Materials in Hebei Province, School of Materials Science and Engineering, Hebei University of Technology, Tianjin 300401, China; hutcui@hebut.edu.cn (C.C.); zhlch@hebut.edu.cn (L.Z.); 4 State Key Laboratory of Materials Processing and Die & Mould Technology, School of Materials Science and Engineering, Huazhong University of Science and Technology, Wuhan 430074, China; pangong@hust.edu.cn

**Keywords:** Fe-rich Al–Si alloy, β-Al_5_FeSi phase, cooling rate, melt spinning, microstructure, strengthening mechanisms

## Abstract

The mechanical properties of iron-rich Al–Si alloy is limited by the existence of plenty of the iron-rich phase (β-Al_5_FeSi), whose unfavorable morphology not only splits the matrix but also causes both stress concentration and interface mismatch with the Al matrix. The effect of the cooling rate on the tensile properties of Fe-rich Al–Si alloy was studied by the melt spinning method at different rotating speeds. At the traditional casting cooling rate of ~10 K/s, the size of the needle-like β-Al_5_FeSi phase is about 80 μm. In contrast, the size of the β-Al_5_FeSi phase is reduced to 500 nm and the morphology changes to a granular morphology with the high cooling rate of ~10^4^ K/s. With the increase of the cooling rate, the morphology of the β-Al_5_FeSi phase is optimized, meanwhile the tensile properties of Fe-rich Al–Si alloy are greatly improved. The improved tensile properties of the Fe-rich Al-Si alloy is attributed to the combination of Fe-rich reinforced particles and the granular silicon phase provided by the high cooling rate of the melt spinning method.

## 1. Introduction

Recycled aluminum is obtained through the recycling of waste aluminum products. The production energy consumption is only 3% to 5% of the energy consumption of primary aluminum [1]. It has obvious advantages in energy saving and emission reduction, thus it has become one of the research hotspots in the field of lightweight structural materials. Iron is considered to be the most harmful impurity element in recycled aluminum. The main reason is that the solid solubility of iron in cast aluminum–silicon alloy is low (only 0.05 wt.%) and a variety of iron-rich intermetallics can be formed during solidification, such as α-AlFeSi, β-Al_5_FeSi, π-Al_8_Mg_3_FeSi_6_, and δ-Al_4_FeSi_2_. Among them, β-Al_5_FeSi is long and needle-like on the two-dimensional optical micrographs and the three-dimensional space is a lath structure [2,3,4].

The size and morphology of the secondary phase are the key factors affecting the properties of the alloy. The needle-like β-Al_5_FeSi phase is easy to cause stress concentration and split the matrix, which is unfavorable to the mechanical properties of recycled aluminum [5,6]. In addition, excessive iron content will lead to poor melt fluidity and reduce casting quality [7]. Therefore, many researchers are working to modify the morphology of β-Al_5_FeSi phases, such as by adding the alloying elements Mn, Nb, Co, Sc, Er, etc. [8,9,10,11,12]. In addition, increasing the melt superheat and heat treatment are also effective ways to improve the morphology of the iron-rich phase. In our previous work, it was found that the combination addition of Mg and La elements can promote the transition from β-Al_5_FeSi to the script-like π-Al_8_Mg_3_FeSi_6_ phase, resulting in a 65% increase in the yield strength of Al–Si–Mg–Fe alloy [13]. However, the addition of the above elements only changes the morphology of the iron-rich phase, brings some technical problems, and further increases the difficulty of the secondary resource recovery of Al–Si alloys. Therefore, it is a key issue to develop high performance Al–Si alloys with a high Fe content.

Increasing the cooling rate is an effective way to change the morphology of an intermetallic. Melt spinning is a typical rapid solidification method which is used in the preparation of inoculants due to its chemical uniformity and large solid solubility [14,15,16]. Additionally, the microstructure evolution has been reported to be dependent on the cooling rate and Fe content in the Al–Si alloy [17,18]. The cooling rates range from 1 to 10 K/s in traditional mold casting to 10 K/s–100 K/s in high-pressure die casting. It can be seen that the morphology control of the cooling rate on the β-Al_5_FeSi phase is generally limited to the sub-rapid solidification (cooling rates < 10^3^ K/s) condition and there is a need to explore the microstructure under the rapid solidification condition with cooling rates higher than 10^3^ K/s. Moreover, although some studies have analyzed the growth behavior and morphology of the needle-like β-Al_5_FeSi phases in specific recycled Al–Si alloy, a systematic way to understand the formation or suppression of the needle-like phase is not available.

In this work, we achieve different cooling rates by adjusting the speed of the copper roller during melt spinning. Through in-depth analysis of the phase microstructure morphology of β-Al_5_FeSi under different cooling rates, the solidification path and formation conditions are discussed. Meanwhile, the effect of the cooling rates on the microstructure and tensile properties using the rapid solidification technology is further investigated.

## 2. Materials and Methods

The Al-10Si-1.5Fe (all compositions cited in this work are in wt.%) alloy investigated in this work was prepared from the as-cast ingots of commercial Al-12Si and Al-20Fe alloys, and its chemical composition is shown in Table 1. The raw and processed materials were melted in a vacuum arc furnace under an argon atmosphere. For a low cooling rate sample, the ingots were remelted by the induction smelting method, during which it was poured into the preheated 200 °C steel mold with a cavity diameter of 20 mm and a height of 120 mm, which was denoted as the C1 alloy. For high cooling rate samples, secondary melting of intermediate alloy was carried out in a high-frequency induction smelting furnace and blown on a copper roller with a diameter of 22 cm and rotating speed of both 2000 rpm and 4000 rpm to produce Al-10Si-1.5Fe ribbons (abbreviated as C2 and C3 alloy, respectively). According to the results of the previous studies [19,20,21,22,23], the cooling rates were calculated as 6.7 × 10^2^ K/s, 2.4 × 10^4^ K/s, respectively.

The specimens were cut from the middle of the casting rod and the spun ribbon for metallographic examination, and then were carefully ground by sandpaper in ethanol as well as dried with air. After mechanical polishing, the specimens were corroded by Poulton’s reagent and observed by optical microscope. The microstructure of the samples was etched with 0.5% vol. HF solution before scanning electron microscope (SEM, Hitachi, Tokyo, Japan) observation. The chemical composition of the phases in the prepared alloys were examined by energy dispersive spectroscopy (EDS, Hitachi, Tokyo, Japan), which was performed on at least 3 parts of the phases. The tensile tests for the as-cast rods were carried out at a constant strain rate of 5 × 10^−4^ s^−1^ and loaded parallel to the axis of the specimens following standard ASTM B209. Based on the same experimental scheme, the C2 and C3 ribbon samples were tensile-tested with a width of 2 mm and length of 120 mm. Three tests were conducted for each sample condition. The samples were characterized by Bruker D8 Discover X-ray diffraction (XRD, Bruker AXS, Karlsruhe, Germany), Cu Ka radiation, the S4800 scanning electron microscope (SEM, TESCAN, Czech Republic), Nova Nano SEM450 (SEM, FEI Company, Portland, America) and the optical microscope (OM, Olympus, Jiang dong ou yi testing instrument, Ningbo, China).

## 3. Results and Discussion

**Figure 1:**
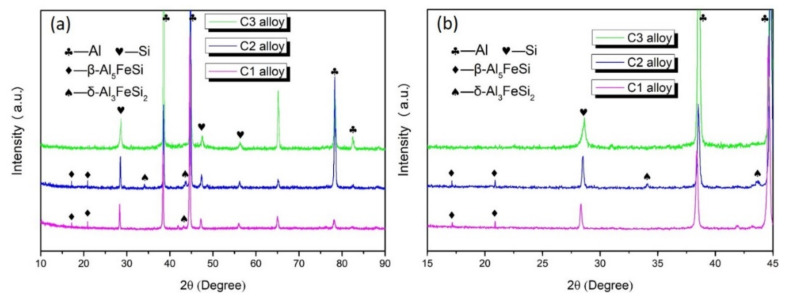
(**a**) XRD spectra of the C1, C2, and C3 alloys. (**b**) Shows the partially amplified spectra of (**a**).

Figure 1 shows the typical XRD spectra of Al-10Si-1.5Fe alloys with different cooling rates. It can be seen that at the cooling rate of ~30 K/s, the C1 alloy had three groups of sharp diffraction peaks corresponding to Al, Si, and β-Al5FeSi phases, respectively. It is clear that the δ-Al_3_FeSi_2_ phase appeared in the C2 alloy with the cooling rate increase to 6.7 × 10^2^ K/s. It is noteworthy that the iron-rich phase disappeared in the matrix as the cooling rate continued to increase to 2.4 × 10^4^ K/s.

**Figure 2:**
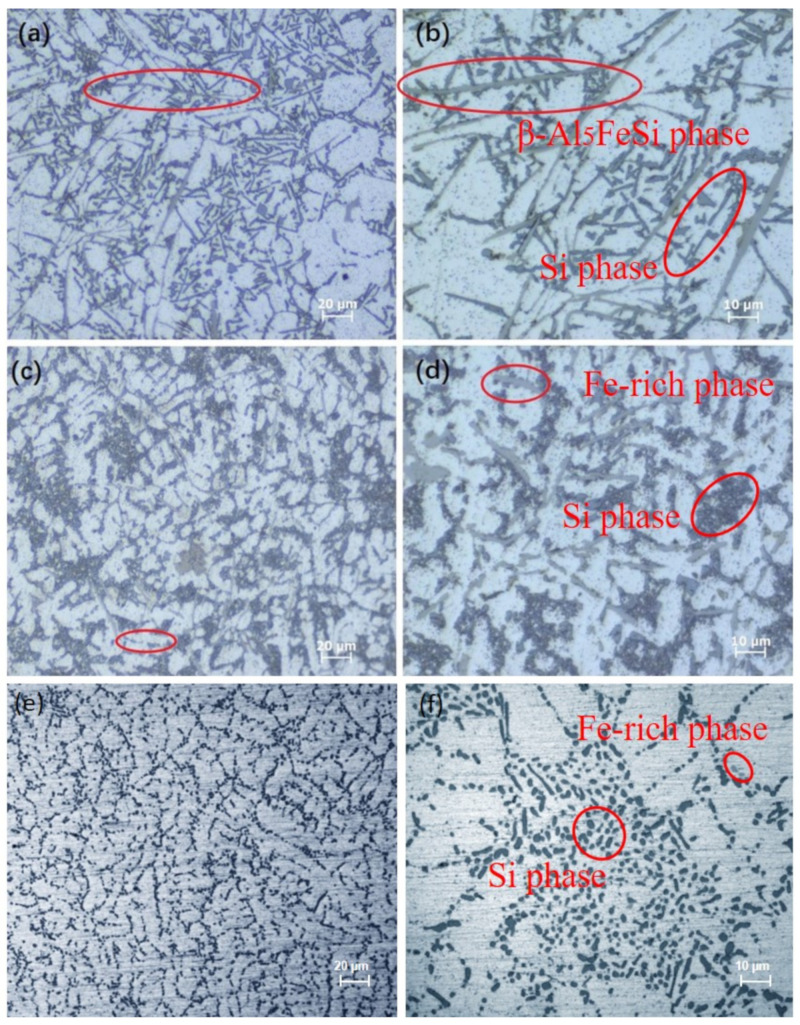
(**a**,**c**,**e**) are optical micrographs of C1, C2, and C3 alloy. (**b**,**d**,**f**) are optical micrographs showing locally amplified spectra of (**a**,**c**,**e**), respectively.

Figure 2 shows the metallography of Al–Si–Fe alloy with different cooling rates. A large number of needle-like phases about 80 μm in length and the acicular-like phase existed in the aluminum matrix. Take the XRD spectra into consideration; they are β-Al_5_FeSi phase and eutectic Si phase, respectively. It can be seen that the β-Al5FeSi phase was randomly distributed among the dendrites, as shown in Figure 2a, and its unfavorable morphology is particularly unfavorable to the strength and ductility of the matrix. It is noteworthy that the morphology of the Fe-rich phase in C2 changed significantly, that is, from needle-like to short rod-like with a length of 10 μm with the increase of the cooling rate to ~10^2^ K/s. In addition, increasing the cooling rate not only changes the morphology of the Fe-rich phase but also the eutectic Si phases transformed from coarse acicular-like to fine particle-like in the C2 alloy. As the cooling rate increased to ~10^4^ K/s, the needle-like or fibrous phases were no longer visible in the field of view and a large number of dispersed particles were replaced, as shown in Figure 2e,f. Compared with the C1 and C2 alloy, the phases distributed in the C3 alloy matrix were more uniform and dispersed.

**Figure 3:**
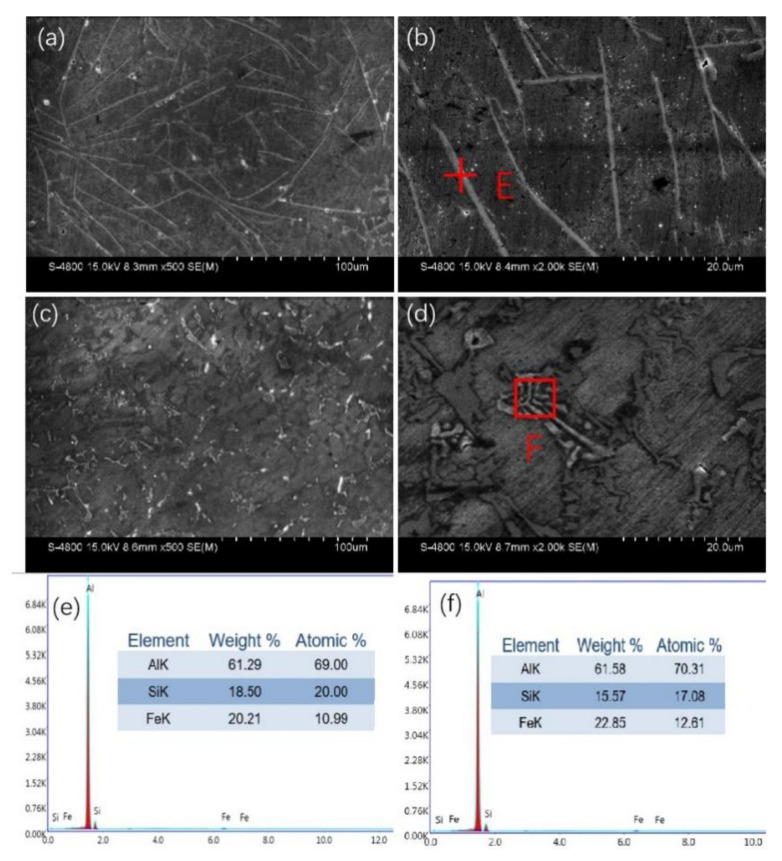
SEM images of the (**a**,**b**) C1 alloy and (**c**,**d**) C2 alloy. (**e**,**f**) are the EDS results of the (**b**,**d**) corresponding areas.

Figure 3 shows the SEM images of Al-10Si-1.5Fe alloys at different cooling rates. Figure 3a,c are the SEM images of the C1 and C2 samples, among which Figure 3b,d are the local enlarged views of Figure 3a,c. In Figure 3a,b, the presence of long needle-like phases and sharp morphology in the die casting sample is more clearly observed than in Figure 3c–f. Combined with the EDS analysis in Figure 3e,f, the phase can be judged as β-Al_5_FeSi. The comparison of Figure 3a,c show that the Fe-rich phase transformed from a sharp long needle-like structure to a short rod-like morphology, wherein the distribution state was more diffuse and there was almost no obvious interweaving connection. Figure 3d clearly shows that the short rod-shaped phase in the C2 alloy with the cooling rate of 6.7 × 10^2^ K/s was a fishbone-like shape, which can be determined as the δ-Al_3_FeSi_2_ phase. The fishbone-like morphology phase had little effect on the fracture of the Al matrix, thus the mechanical properties of the matrix will be improved accordingly. Based on the above analysis, the increase in the cooling rate does have a regulatory effect on the morphology of the Fe-rich phase in the aluminum alloy.

**Figure 4:**
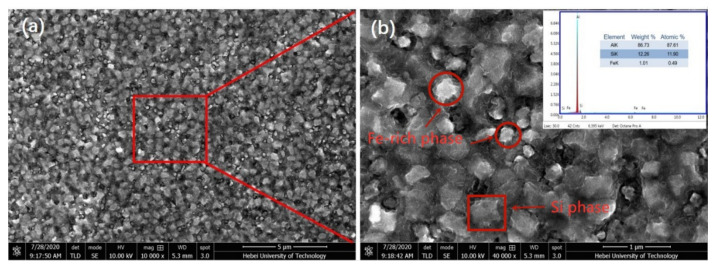
(**a**) SEM images of the C3 alloy. (**b**) is the magnification of the region in (**a**), while the inset is the EDS results of the region in (**a**).

**Figure 5:**
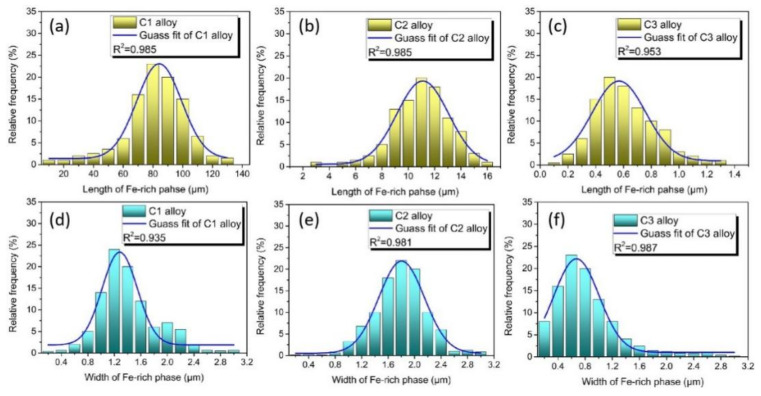
Statistical distribution of the length of Fe-rich phase of the (**a**) C1, (**b**) C2, and (**c**) C3 alloys, (**d,e,f**) are the statistical distribution of the width of Fe-rich phase of the C1, C2 and C3 alloys, respectively.

Figure 4 reveals the SEM images of the C3 alloy with the cooling rate of 2.4 × 10^4^ K/s. It can be seen that two kinds of particles with different brightness were distributed in the matrix and the particle size ranged from 200 nm to 500 nm. From the EDS inset in Figure 4b, it can be seen that the content of iron in this region was about 1 wt.%, which is lower than the nominal composition of the Al–Si–Fe alloy. The cooling rate can obviously change the morphology of iron-rich phase because at a high cooling rate, Fe does not have enough time to precipitate in the form of the intermetallic phase. Instead, Fe is trapped in the Al matrix to form a supersaturated solid solution. In order to clearly describe the change of the Fe-rich phase size with the cooling rate, the particle length and width statistical distribution of the three alloys is shown in Figure 5. It can be seen that with the increase of the cooling rate, the length of the Fe-rich phase decreased sharply and the length and width tended to be consistent, which tends to form spherical particles. The change is beneficial to the improvement of the tensile properties of the Fe-rich Al alloy.

**Figure 6:**
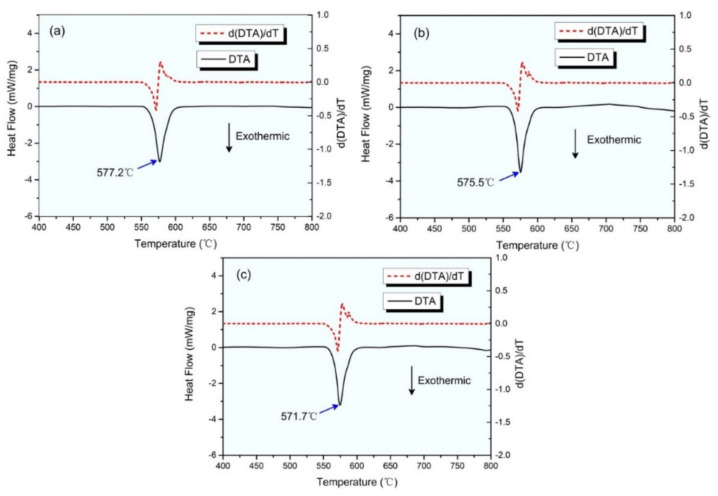
(**a**–**c**) DTA thermograph of C1 alloy, C2 alloy, and C3 alloy, respectively.

Figure 6 reveals the Differential Thermal Analysis (DTA)curves of the alloy at different cooling rates, where the peak of the DTA curve represents the eutectic temperature of the alloy. As the cooling rate increased, the eutectic temperature gradually decreased from 577.2 °C to 571.7 °C. According to the binary phase diagram of Al–Si alloy, the eutectic zone moves towards the non-metallic phase under the non-equilibrium solidification condition. The growth morphology of the silicon phase is not only restricted by the concentration distribution of the silicon atom at the growth interface but also is affected by the growth rate of α-Al. When the cooling rate is high, the α-Al phase is the first to nucleate and grow during solidification, and silicon atoms are disposed to the front of the solid–liquid interface, which increases the concentration of the silicon element in liquid phase. At this time, the silicon phase changes its growth direction in staggered or twin mode. It can be seen that from the C2 and C3 alloys, they had a subtle derivative curve peak in the eutectic region corresponding to the formation of the δ-Al_3_FeSi_2_ phase. That is, an increase in the cooling rate can promote the precipitate of the fishbone-like δ-Al_3_FeSi_2_ phase to replace the harmful β-Al_5_FeSi phase.

**Figure 7:**
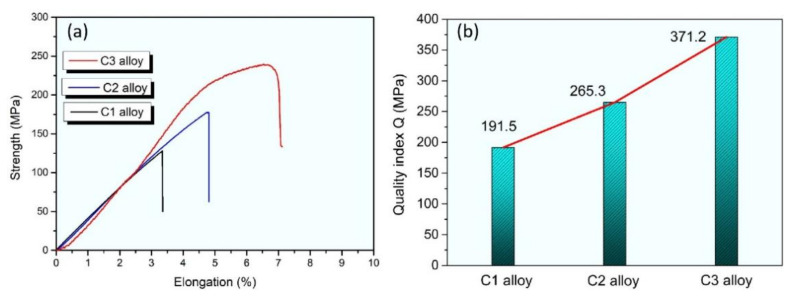
(**a**) Tensile properties of the alloys with different cooling rates. (**b**) The quality index Q of the three alloys.

Figure 7 shows the tensile properties of the alloys with different cooling rates. As the cooling rates increased, the tensile strength and elongation of the alloys were significantly improved, as shown in Figure 7a. The quality index Q can be expressed as follows [24]:

(1) $$Q=\mathrm{UTS}\left(\mathrm{MPa}\right)+150\times \;\log\left(\%\mathrm{EI}\right)$$

Mass coefficient Q includes both tensile strength (UTS) and elongation after fracture (EI), which is a more widely used and reliable parameter in engineering applications. As can be seen from Figure 7, there was no significant difference between the tensile properties of the C2 alloy and the as-cast C1 alloy, which is consistent with the results of Figure 2 and Figure 3. The morphology of the needle-like β-phase did not change completely at the moderate cooling rate but the aspect ratio decreased. However, the tensile properties of the C3 alloy with the high cooling rate are obviously improved compared with those of the C1 alloy and C2 alloy. It is mainly attributed to the transformation of the coarse needle-like β-phase into the δ-Al_3_FeSi_2_ granular phase. In addition, the quality index of the C3 alloy is obviously higher than those of the other two alloys. The other main reason is that the acicular eutectic silicon phase transforms into fine fibers, which significantly reduces the stress concentration around the acicular silicon phase during the tensile process. As the cooling rates increased, this remarkably reduced the aspect ratio of the silicon phase. Overall, the strengthening mechanism is considered to be associated with the morphology change of the needle-like β-phase and with the decrease in aspect ratio of the eutectic silicon phase. The optimization of the phase morphology can significantly reduce the stress concentration and improve the load transfer capacity so as to inhibit the initiation and propagation of cracks during the tensile process [25,26,27].

**Figure 8:**
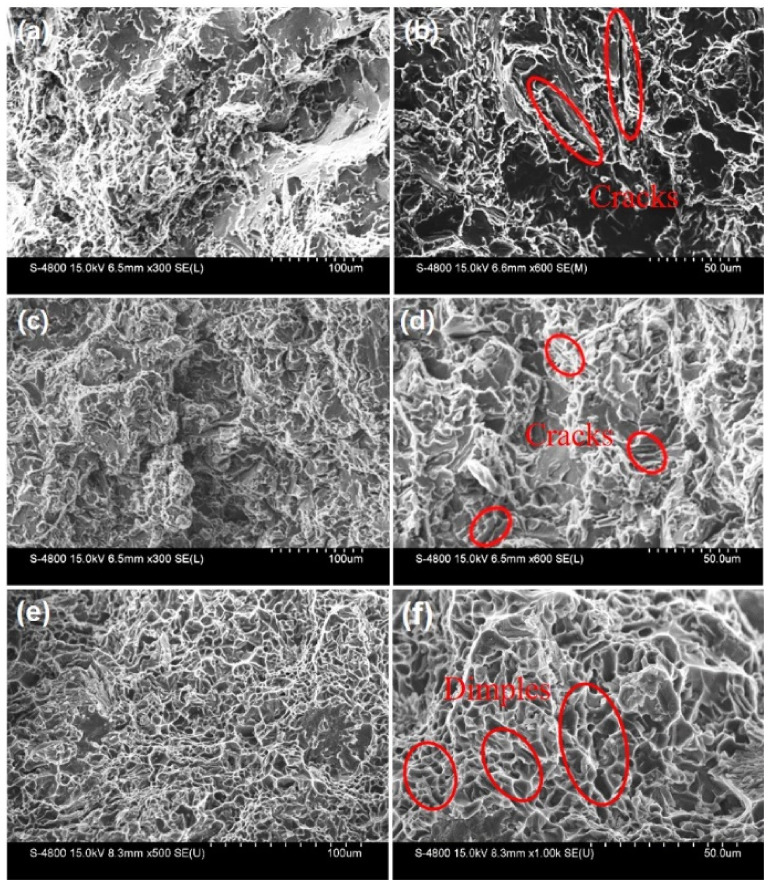
Fracture surface of the alloys with different cooling rates: (**a**,**c**,**e**) represent C1, C2, and C3 alloy, respectively. (**b**,**d**,**f**) are the enlarged images of the corresponding areas.

In order to better understand the failure mechanism of the alloy at different cooling rates, fracture studies were carried out on each tensile fracture sample. Figure 8a,c show the cleavage mode of the fracture with evidence of many brittle cleavage planes separated by tearing ridges. It can be observed in the high-magnification SEM fractography of Figure 8a,c that coarse secondary phases caused the initiation and fast propagation of cracks. This can be responsible for the low elongation value of the alloys. The plastic deformation of α-Al grains are inhibited because the dislocation cannot pass through the β phase by the shear or bypass mechanism [28,29]. As a result, dislocations continue to accumulate at the interface between the β-phase and aluminum matrix and result in severe stress concentrations, which lead to a higher tendency of brittle fractures. The existence of these cleavage planes indicates that these alloys exhibit brittle fracture and need sufficient modification for practical applications. It is worth noting that the fracture mode of the C3 alloy was different from that of the C1 and C2 alloys, and a large number of dimples appeared on the fracture surface, as shown in Figure 8e,f. The ductile fracture was determined by the size of the dimples, meaning that more homogenous and deeper dimples produce a higher ductility of the alloys. The existence of deep and conical dimples in the C3 alloy shows the higher ductility, which is consistent with the results shown in Figure 7. The realization of better mechanical properties is attributed to the combined effect of the harmful morphology of the β phase and to the spheroidization of the silicon phase.

## 4. Conclusions

(1) The morphology of the Fe-rich phase in Al–Si alloy is greatly affected by the cooling rate. The needle-like β-Al_5_FeSi phase had the size of ~80 μm at the conventional casting cooling rate. As the cooling rate increases to ~10^2^ K/s, the needle-like β-phase changes into the fishbone-like δ-Al_3_FeSi_2_ phase. At a high cooling rate of ~10^4^ K/s, the size of the β-Al_5_FeSi phase decreases to ~500 nm and the morphology changes to a granular form.

(2) The change of cooling rate reduces the eutectic temperature of Fe-rich Al–Si alloy and promotes the phase transformation of eutectic silicon. The DTA analysis results showed that the increase in the cooling rate contributes to forming the fishbone-like δ-Al_3_FeSi_2_ phase and plays an important role in modifying the structure of the eutectic Si.

(3) The platelet-like β phase is indeed the initiation point of the crack, which can be confirmed by the fracture morphology analysis. In addition, the platelet-like β-phase accelerates crack propagation. On the contrary, the fine Fe-rich particles are relatively helpful to improve the tensile strength and elongation.

(4) The melt spinning method transforms the brittle failure of Fe-rich Al–Si alloy into ductile failure, which is of great significance to the recovery and reuse of Fe-rich Al–Si alloy and has broad industrial application prospects.

## Figures and Tables

**Table 1:** Composition of the experimental alloy.

Element	Al	Si	Fe	Mg	Cu	Zn
Content	Balance	9.96	1.52	0.33	0.09	0.07

## Data Availability

The data used to support the findings of this study are available from the corresponding author upon request.

## References

[1] Zhu X., Blake P., Dou K., Ji S. (2018). Strengthening die-cast Al-Mg and Al-Mg-Mn alloys with Fe as a beneficial element. Mater. Sci. Eng. A.

[2] Puncreobutr C., Lee P.D., Karech K.M., Connolley T., Fife J.L., Phillion A.B. (2014). Influence of Fe-rich intermetallics on solidification defects in Al-Si-Cu alloys. Acta Mater..

[3] Cameron M.D., John A.T., Arne K.D. (2005). As-cast morphology of iron-intermetallics in Al-Si foundry alloys. Scripta Mater..

[4] Terzi S., Taylor J.A., Cho Y.H., Salvo L., Suery M., Boller E., Dahle A.K. (2010). In situ study of nucleation and growth of the irregular α-Al/β-AlFeSi eutectic by 3D synchrotron X-ray microtomography. Acta Mater..

[5] Feng S.K., Liotti E., Lui A., Matthew D., Connolley T., Mathiesen R., Patrick S. (2020). In-situ X-ray radiography of primary Fe-rich intermetallic compound formation. Acta Mater..

[6] Que Z.P., Wang Y., Zhang F. (2018). Formation of the Fe-containing intermetallic compounds during solidification of Al-5Mg-2Si-0.7Mn-1.1Fe alloy. Metall. Trans. A..

[7] Gao T., Li Z.Q., Zhang Y.X., Qin J.Y., Liu X.F. (2017). Evolution of Fe-rich phases in Mg melt and a novel method for separating Al and Fe from Al-Si-Fe alloys. Mater. Des..

[8] Li X.F., Xia C.J., Wu Y., Chen D., Wang M.L., Ma N.H., Wang H.W. (2019). Effect of Er addition on the high temperature strength of Al-Si-Cu-Ni-Mg-Fe piston alloys by T5 and T6 heat treatment. Mater. Sci..

[9] Wu X.Y., Zhang H.R., Zhang F.X., Ma Z., Yang B., Tao T.X., Zhang H. (2018). Effect of cooling rate and Co content on the formation of Fe-rich intermetallics in hypoeutectic Al7Si0.3Mg alloy with 0.5%Fe. Mater. Charact..

[10] Tang Q., Zhao J.H., Chen J., He K. (2018). The effects of neodymium addition on the intermetallic microstructure and mechanical properties of Al-7Si-0.3Mg-0.3Fe alloys. J. Alloys Compd..

[11] Zhang J.Y., Feng J., Zuo L.J., Ye B., Kong X.Y., Jiang H.Y., Ding W.J. (2019). Effect of Sc microalloying addition on microstructure and mechanical properties of as-cast Al-12Si alloy. Mater. Sci. Eng. A.

[12] Khan M.H., Das A., Li Z., Kotadia H.R. (2021). Effects of Fe, Mn, chemical grain refinement and cooling rate on the evolution of Fe intermetallics in a model 6082 Al-alloy. Intermetallics.

[13] Li D.F., Cui C.X., Wang X., Wang Q.Z., Chen C., Liu S.Q. (2016). Microstructure evolution and enhanced mechanical properties of eutectic Al-Si die cast alloy by combined alloying Mg and La. Mater. Des..

[14] Easton M.A., StJohn D.H. (2008). Improved prediction of the grain size of aluminum alloys that includes the effect of cooling rate. Mater. Sci. Eng. A.

[15] Verma A., Kumar S., Grant P.S., O’Reilly K.A.Q. (2013). Influence of cooling rate on the Fe intermetallic formation in an AA6063 Al alloy. J. Alloys Compd..

[16] Kilicaslan M.F., Altaib S.S., Vurdu C.D. (2019). Effect of Ni Addition on the Morphology and Microstructure of Both Conventional Cast and Melt-Spun of Al-Si-Fe-Nb (at wt%) Alloy. Met. Mater. Int..

[17] Rajabi M., Simchia A., Davamia P. (2008). Microstructure and mechanical properties of Al-20Si-5Fe-2X (X=Cu, Ni, Cr) alloys produced by melt-spinning. Mater. Sci. Eng. A.

[18] Liu Z.T., Wang B.Y., Wang C., Zha M., Liu G.J., Yang Z.Z., Wang J.G., Li J.H., Wang H.Y. (2020). Microstructure and mechanical properties of Al-Mg-Si alloy fabricated by a short process based on sub-rapid solidification. J. Mater. Sci. Technol..

[19] Liu S.Q., Wang X., Cui C.X., Zhao L.C., Liu S.J., Chen C. (2015). Fabrication, microstructure and refining mechanism of in situ CeB6/Al inoculant in aluminum. Mater. Des..

[20] Liu S.Q., Wang X., Cui C.X., Zhao L.C. (2017). Enhanced grain refinement of in situ CeB_6_/Al composite inoculant on pure aluminum by microstructure control. J. Alloys Compd..

[21] Liu S.Q., Wang X., Cui C.X., Han X., Cui C. (2021). Significantly improved particle strengthening of Al-Sc alloy by high Sc composition design and rapid solidification. Mater. Sci. Eng. A.

[22] Liu S.Q., Cui C.X., Wang X., Li N., Cui S. (2017). Effect of cooling rate on microstructure and grain refining behavior of in situ CeB_6_/Al composite inoculant in aluminum. Metals.

[23] Han B.H., Liu S.Q., Wang X., Cui C.X. (2021). Simultaneously improving strength and ductility of hybrid Al-Si matrix composite with polyphasic and multi-scale ceramic particles reinforced. Mater. Sci. Eng. A.

[24] Wu X.F., Wang Z.C., Wang K.Y., Zhao R.D., Wu F.F. (2022). Microstructural refinement and tensile properties enhancement of Al-10Mg2Si cast alloys by copper addition. J. Alloys Compd..

[25] Azimi H., Nourouzi S., Jamatti R. (2021). Effects of Ti particles and T6 heat treatment on the microstructure and mechanical properties of A356 alloy fabricated by compocasting. Mater. Sci. Eng. A.

[26] Liu T., Pei Z.R., Barton D., Brewer L.N. (2022). Characterization of nanostructures in a high pressure die cast Al-Si-Cu alloy. Acta Mater..

[27] Bogdanoff T., Lattanzi L., Merlin M., Ghassemali E., Jarfors A.E.W., Senfeddine S. (2021). The complex interaction between microstructural features and crack evolution during cyclic testing in heat-treated Al–Si–Mg–Cu cast alloys. Mater. Sci. Eng. A.

[28] Kim D., Kim J.H., Jeon J.Y., Kim Y.W., Kobayashi E. (2021). Local elongation of a high Fe-containing Al-Si-Cu-Mg alloy by a deformation-semisolid extrusion process. Mater. Lett..

[29] Que Z.P., Mendis C.L. (2020). Heterogeneous nucleation and phase transformation of Fe-rich intermetallic compounds in Al–Mg–Si alloys. J. Alloys Compd..

